# A STRUCTURED APPROACH TO EVALUATING LIFE COURSE INFLUENCES ON DNA METHYLATION AGE ACCELERATION

**DOI:** 10.1093/geroni/igad104.0721

**Published:** 2023-12-21

**Authors:** Jessica Faul, Stacey Collins, Mateo Farina, Eric Klopack, Zainab Masood, Helen Meier, Colter Mitchell, Eileen Crimmins

**Affiliations:** University of Michigan, Ann Arbor, Michigan, United States; University of Michigan, Ann Arbor, Michigan, United States; University of Texas at Austin, Austin, Texas, United States; University of Southern California, Los Angeles, California, United States; University of Michigan, Ann Arbor, Michigan, United States; University of Michigan, Ann Arbor, Michigan, United States; University of Michigan, Ann Arbor, Michigan, United States; University of Southern California, Los Angeles, California, United States

## Abstract

Lower socio-economic position (SEP) is associated with increased biological age acceleration measured using DNA methylation (DNAm). Prior studies using cohorts from the US and UK have shown a relationship between social mobility and DNAm age acceleration such that those who experienced upward mobility exhibited slower biological aging relative to continuously advantage groups while those with disadvantaged SEP across the life course experienced the most rapid DNAm age acceleration. However, using a social mobility model, it is not clear to what extent childhood may be a sensitive period for the impact of social disadvantage on DNA methylation or whether an accumulation of disadvantage hypothesis is consistent with the data. In this study, we apply a structured life-course modeling approach (SLCMA), a theory-driven analytical method that compares life course hypotheses of time-dependent exposure-outcome relationships, to determine whether early life is a sensitive period for DNAm modification using data from approximately 4,000 participants of the 2016 Health and Retirement Study (HRS) Venous Blood Study. We hypothesize that childhood is a sensitive period during which exposure to lower socio-economic position results in greater DNAm changes. We compare this to alternative hypotheses including the accumulation of disadvantage, in which the effect of low SEP increases with the duration of exposure, and the recency of disadvantage, in which the effect of low SEP is stronger for exposure in later life.

